# Cyclophosphamide in combination with tumor lysate and TLR9 ligand regresses murine neuroblastoma

**DOI:** 10.1186/2051-1426-1-S1-P74

**Published:** 2013-11-07

**Authors:** Jill Gershan, Kristen Barr, James Weber, Bryon Johnson

**Affiliations:** 1 Pediatrics, Medical College of Wisconsin, Milwaukee, WI, USA

## 

Neuroblastoma is a pediatric cancer of neural crest origin that accounts for 12% of all pediatric cancer deaths. Despite improved survival in patients that receive standard care plus anti-GD2 immune therapy, there remains a 40% mortality rate in high-risk patients. This high mortality emphasizes the need for continued testing of immune-based therapies for the treatment of refractory neuroblastoma. Using a murine model, we previously observed a cell-mediated immune response resulting in elimination of established neuroblastoma when mice were given a whole-cell tumor vaccine expressing CD80, CD86, CD54 and CD137L. Given the anti-tumor efficacy of this immune-based therapy, we designed and tested a more clinically translatable protocol for the treatment of established neuroblastoma. In this study, unmodified tumor cell lysate and the TLR9 ligand, CpG-ODN 1826 (CpG), was given as immune therapy in combination with cyclophosphamide (CY). To establish tumor burden, mice were inoculated subcutaneously (s.c.) with 10E5 live neuroblastoma cells. Four and five days following tumor inoculation mice received 100 mg/kg intravenous cyclophosphamide. Nine 16, 23, and 30 days following tumor inoculation some mice received the tumor cell lysate and CpG immune therapy. When only CY was administered, all mice succumbed to tumor. When tumor lysate and CpG were added to the CY treatment protocol, tumors regressed and there was 60% survival. Immune cell analysis revealed an increase in activated mature (I-Ak+CD11c+) dendritic cells in the spleen and draining lymph nodes of mice that received CY, CpG and tumor lysate. We also found evidence of an adaptive immune response. Mice treated with CY, CpG and tumor lysate had an increase in the percentage of Ki67+ proliferating splenic CD4 and CD8 T cells. In addition, there was an increase in T effector memory (CD44hiCD62Llo) cells in the spleens of mice treated with Cy, lysate and CpG. Splenic T cells harvested from these mice produced interferon gamma (IFN-γ) when exposed to neuroblastoma tumor cells. Of great importance for clinical translation, when either autologous or allogeneic lysate was given in combination with CY and CpG, IFN-γ was produced in tumor-specific splenic CD8 T cells. Survival curves were similar regardless of administration of autologous or allogeneic tumor lysate. We are continuing to explore the treatment-related immune mechanisms that promote tumor regression with hope for clinical translation of this protocol.

